# Associations of retinol-binding protein 4 with oxidative stress, inflammatory markers, and metabolic syndrome in a middle-aged and elderly Chinese population

**DOI:** 10.1186/1758-5996-6-25

**Published:** 2014-02-24

**Authors:** Yan Liu, Duan Wang, Di Li, Ruifang Sun, Min Xia

**Affiliations:** 1 Guangdong Provincial Key Laboratory of Food, Nutrition and Health, Guangzhou, P.R. China; 2 Department of Nutrition, School of Public Health, Sun Yat-sen University (Northern Campus), Guangzhou, Guangdong Province 510080, P.R. China

**Keywords:** RBP4, Oxidative stress, Inflammatory markers, Diabetes mellitus

## Abstract

**Background:**

Retinol-binding protein 4 (RBP4), a novel adipokine secreted by adipocytes and the liver, has elevated levels in type 2 diabetes mellitus (T2DM). However, its association with human metabolic diseases remains controversial. The present study was designed to investigate the associations of plasma RBP4 levels with oxidative stress, inflammatory markers, and metabolic syndrome (MetS) in a Chinese population.

**Method:**

We evaluated plasma RBP4 levels in a cross-sectional sample of 1748 Chinese men and women aged 50 to 70 years in Guangzhou using an in-house developed and validated sandwich ELISA. Plasma glucose, insulin, lipid profile, serum adiponectin, adipocyte fatty acid-binding protein (A-FABP), 8-iso-prostaglandin F2α (8-iso PGF2α), 13-(S)-hydroxyoctadecadienoic acid (13-HODE), high-sensitivity C-reactive protein (hsCRP), interleukin 6 (IL6), monocyte chemotactic protein 1 (MCP1) and tumor necrosis factor α (TNFα) were all measured. MetS was defined according to the updated National Cholesterol Education Program Adult Treatment Panel III criteria for Asian Americans.

**Results:**

Circulating RBP4 levels were positively correlated with A-FABP (*r* = 0.104, *P* < 0.001), 8-iso PGF2α (0.236, *P* < 0.001), and 13-HODE (0.204, *P* < 0.001) and were inversely correlated with HDL cholesterol (*r* = −0.072, *P* = 0.004). After multivariable adjustment, the RBP4 levels were strongly associated with MetS and its components. The ORs (95% CIs) for the comparisons of the extreme quartiles of RBP4 were 3.46 (2.87, 4.42) for MetS, 5.92 (4.47, 8.02) for hypertriglyceridemia, 1.42 (1.11, 1.68) for reduced HDL cholesterol, 1.87 (1.48, 2.36) for central obesity and 2.74 (2.15, 3.36) for hyperglycemia (all *P* < 0.001). When we further controlled for adipokines, markers of oxidative stress and proinflammatory response, the association of RBP4 with central obesity was abolished but not the association with other MetS components.

**Conclusions:**

Plasma RBP4 levels are associated with an adverse profile of oxidative stress and inflammatory markers and an increased risk of MetS in this Chinese population. These associations are independent of conventional risk factors.

## Introduction

Metabolic syndrome (MetS) is a clustering of multiple metabolic abnormalities, including central obesity, dyslipidemia, elevated blood pressure, hyperglycemia and insulin resistance. MetS plays an important role in the origin of cardiometabolic diseases, including cardiovascular disease (CVD) and type 2 diabetes mellitus [1-3]. Multiple mechanisms may contribute to MetS development, including an abnormal production of adipocyte- secreted proteins (adipocytokines), aberrant oxidative stress and dysregulated proinflammatory responses in tissues such as the muscle and liver [4-7]. Therefore, it is of vital importance to identify key risk factors for the early diagnosis and intervention of metabolic diseases.

Retinol-binding protein 4 (RBP4), mainly secreted by adipocytes and the liver, was originally known as the specific carrier of retinol in circulation [8]. Recent studies demonstrated that RBP4 levels were increased in obese and insulin-resistant humans and mouse models; additionally, a genetic or pharmacologic elevation of serum RBP4 causes insulin resistance in normal mice [9-11]. Although many studies show strong correlations of serum RBP4 levels with the severity of insulin resistance and obesity and with certain components of MetS, including hypertension [12], dyslipidemia [12,13], waist/hip ratio [13], cardiovascular disease [14,15], and intra-abdominal fat mass [16], others do not demonstrate these correlations [17-19]. These inconsistencies may result from differences in age, ethnicity, population size, and the methodological techniques used [20]. Hence, it has yet to be established whether RBP4 may serve as a risk marker for insulin resistance and type 2 diabetes and to what extent it is associated with MetS.

Therefore, we examined the association between RBP4 and MetS risk among a middle-aged and elderly Chinese population. We focused on the correlation of RBP4 with oxidative stress and inflammatory factors, both of which are established or proposed risk factors for metabolic disorders.

## Methods

### Study participants

The study population consisted of individuals who underwent the Nutrition and Health of Aging Populations in South China study, which investigated the associations of dietary and genetic factors, as well as their interactions, with aging-related chronic diseases. From March 2008 to March 2009, 2289 participants aged 50–70 years were recruited from Guangzhou if they had been residents in their respective cities for 10 years. For this study, participants were excluded if one or more of the following criteria were met: younger than 50 years old; had a history of cancer, diabetes, CVD, or stroke; or had missing data on one component of the MetS diagnostic. The study protocol was approved by the Institutional Review Board of the Sun Yat-sen University, and written informed consent was obtained from all participants.

### Data collection

Baseline data were collected by trained interviewers via semi-structured questionnaires during face-to-face interviews. The questionnaire was designed based on the pilot surveys among this population. Information on socio-demographic factors, health status, and lifestyle practices (including dietary factors and physical activity) was included in the questionnaire. Standing height, body weight, and waist circumference were measured with the participants in light indoor clothing and without shoes. Body mass index was calculated as weight in kilograms divided by height in meters squared. Two researchers independently entered the baseline data from the questionnaires, and the data were further checked by a third researcher when differences were found. All of the subjects were examined in the morning after an overnight fast.

### Laboratory measurements

Overnight fasting blood samples were collected in tubes containing liquid EDTA, centrifuged at 4°C, and stored at −80°C until analysis. Total blood cholesterol, high-density lipoprotein (HDL) cholesterol, triglycerides and glucose were measured enzymatically on a Hitachi 7180 Biochemistry Automatic Analyzer (Hitachi, Japan) using a commercial assay kit (Wako Pure Chemical Industries, Osaka, Japan). Low-density lipoprotein (LDL) cholesterol was subsequently calculated using the Friedewald formula [21]. Plasma insulin concentrations were measured by a radioimmunoassay (Roche, Indianapolis, IN) that has less than 0.2% cross-reactivity with proinsulin. A homeostasis model assessment of insulin resistance (HOMA-IR) was calculated as fasting insulin (in lIU/mL) × fasting glucose (in mmol/L)/22.5. Serum A-FABP concentrations were measured using an enzyme-linked immunosorbent assay (Cayman, Ann Arbor, MI), and the intra- and interassay CVs were 3.3–7.1% and 2.1–5.7%, respectively.

The serum RBP4 concentrations were measured using an enzyme-linked immunosorbent assay (AdipoGen, Seoul, Korea), and the intra- and interassay CVs were 1.92–3.68% and 6.57–8.59%, respectively. Serum adiponectin concentrations were measured using an enzyme-linked immunosorbent assay (AdipoGen, Seoul, Korea), and the intra- and interassay CVs were 4.1–5.9% and 3.7–6.3%, respectively. Plasma RBP4 levels were measured in duplicate in plasma aliquots that had undergone 1 or 2 freeze-thaw cycles using a competitive enzyme-linked immunosorbent assay (ELISA) according to the manufacturer’s instructions with purified human RBP4 standards (Adipogen, Inc.). The ELISA samples were run in duplicate. The coefficient of variation for interassay replicate samples was less than 7%. The assay system was subsequently cross-validated by a Western blot analysis. The intraassay coefficient of variation was 1.8–7.6%, and that of the interassay was 3.7–8.8%. Plasma 8-iso PGF2α levels were quantitatively determined using an ELISA kit purchased from Enzo Life Sciences International (Plymouth Meeting, PA, USA). The assay sensitivity was 16.3 pg/ml (range 6.1–100,000 pg/ml). The intraassay coefficient of variation was 4.4–11%, and that of the interassay was 5.0–11%. Plasma 13-HODE concentrations were measured using a colorimetric competitive enzyme immunoassay kit (Enzo Life Sciences International). The assay sensitivity was 1.6 pmol/ml (range 0.7–100 pmol/ml). The intraassay coefficient of variation was 6.4–7.3%, and that of the interassay was 5.4–11.3%.

Plasma CRP was measured using a particle-enhanced immunoturbidimetric assay (Ultrasensitive CRP kit, CRM diagnostic systems, Spain) with microparticles coated with anti-human CRP antibodies. The precision of the method in the cut-off value of decision (1.8–2 μg/ml) is less than 5.5%. The serum levels of interleukin 6 (IL6), monocyte chemotactic protein 1 (MCP1), and tumor necrosis factor alpha (TNFα) were measured using a MILLIPLEX™ Human Cytokine/Chemokine panel (Millipore, Billerica, MA). The intra- and interassay coefficients of variation were 8.1 and 11.6% for IL6, 6.1 and 12.0% for MCP1, and 10.5 and 15.9% for TNFα, respectively.

### Definition of MetS

MetS was defined using the updated National Cholesterol Education Program/Adult Treatment Panel III criteria for Asian Americans as having ≥3 of the following components: waist circumference ≥90 cm for men or ≥80 cm for women; triglycerides (TG) ≥1.7 mmol/L; high-density lipoprotein (HDL) cholesterol <1.03 mmol/L for men or <1.30 mmol/L for women; blood pressure ≥130/85 mmHg or current use of antihypertensive medications; or fasting glucose ≥5.6 mmol/L [22].

### Statistical analysis

The normally distributed data were expressed as the means ± SD, whereas variables with a skewed distribution were reported as the median (interquartile range) and log transformed to approximate normality before analysis. The categorical variables were represented by frequency and percentage. An analysis of covariance for the continuous variables and a multivariate logistic regression analysis for the categorical variables were applied for the comparison according to the RBP4 quartiles. An analysis of covariance was used to compare the RBP4 levels between genders and geographic locations. Correlation coefficients between RBP4 and metabolic features were calculated by a partial correlation analysis on ranks (Spearman correlation). The plasma RBP4 levels were depicted according to the number of MetS components using a linear regression model. Multivariate logistic regression models were used to estimate the odds ratios (ORs) for MetS and its components. Potential confounding variables, including age, gender, smoking, alcohol drinking, physical activity, educational level, self-reported CVD, family history of diabetes and CVD, CRP, adiponectin, homeostatic model assessment of insulin resistance (HOMA-IR), and body mass index (BMI), were controlled for in the regression models. The data management and statistical analyses were performed with SPSS 16.0 for Windows (SPSS Inc., Chicago, USA). A *P* < 0.05 was considered statistically significant.

## Results

A total of 1748 individuals (men and women) were included in this study. The characteristics of the study population according to the quartiles of plasma RBP4 concentrations are summarized in Table 1. Because no significant differences by sex were observed, men and women were analyzed together. There were significant associations between plasma RBP4 concentrations and BMI and waist circumference: the participants in the highest quartile of RBP4 had a higher BMI and waist circumference than those in the lower quartiles of RBP4, after adjustment for age and sex (*P* < 0.001). However, no significant associations were observed between RBP4 and current drinking or smoking status.

**Table 1:** Characteristics of the study participants according to RBP4 quartiles

	**Quartile of RBP4**			
	**1 (*n*** **= 437)**	**2 (*n*** **= 437)**	**3 (*n*** **= 437)**	**4 (*n*** **= 437)**
RBP4 (μg/ml)	20.94 ± 4.39^1^	27.97 ± 2.64	33.71 ± 3.05	46.18 ± 8.11
Age (years)	63.89 ± 5.80	65.08 ± 4.81	65.77 ± 4.87	66.23 ± 4.77
Male [*n* (%)]	236 (54.0)	225 (51.5)	230 (52.6)	219 (50.1)
BMI (kg/m^2^)	23.49 ± 2.69	24.26 ± 2.86	24.42 ± 3.37	24.71 ± 3.16
Current smoker [*n* (%)]	109 (24.9)	117 (26.8)	115 (26.3)	122 (27.9)
Current drinker [*n* (%)]	104 (23.8)	98 (22.4)	94 (21.5)	101 (23.1)
Family history of chronic diseases [*n* (%)]^2^	127 (29.1)	117 (26.8)	129 (29.5)	121 (27.7)
Waist circumference (cm)	79.86 ± 10.65	81.48 ± 12.13	84.03 ± 11.75	85.73 ± 12.38
Waist-to-hip ratio	0.84 ± 0.09	0.87 ± 0.07	0.90 ± 0.08	0.92 ± 0.07
Systolic blood pressure (mmHg)	116.81 ± 12.01	115.26 ± 11.96	116.44 ± 11.54	116.56 ± 12.41
Diastolic blood pressure (mmHg)	73.25 ± 8.23	73.88 ± 7.47	74.15 ± 8.42	74.36 ± 8.68
Fasting glucose (mmol/l)	5.62 ± 1.44	5.81 ± 1.93	5.76 ± 1.34	6.02 ± 2.03
Insulin (μU/ml)	13.17 (9.76–16.85)^3^	13.32 (9.53–17.74)	13.85 (10.08–18.21)	14.36 (10.46–19.82)
HOMA-IR	3.29 (2.44–4.21)	3.44 (2.46–4.58)	3.55 (2.58–4.66)	3.84 (2.80–5.31)
Adiponectin (μg/ml)	15.18 (10.87–23.86)	13.54 (9.25–22.35)	13.79 (8.42–20.89)	13.68 (7.83–19.14)
A-FABP (μg/l)	15.38 (8.45–21.91)	15.61 (8.84–21.38)	15.85 (8.66–23.04)	16.12 (9.62–23.42)
Total cholesterol (mM)	4.67 ± 0.78	4.78 ± 0.72	4.89 ± 0.71	5.00 ± 0.58
Triglyceride (mM)	1.04 ± 0.39	1.13 ± 0.36	1.25 ± 0.42	1.35 ± 0.45
LDL cholesterol (mM)	3.06 ± 0.65	3.18 ± 0.61	3.34 ± 0.62	3.50 ± 0.56
HDL cholesterol (mM)	1.48 ± 0.33	1.42 ± 0.34	1.37 ± 0.29	1.33 ± 0.44
8-iso PGF2-α, pmol/ml	7.42 ± 2.56	7.63 ± 2.49	8.54 ± 2.77	9.32 ± 2.89
13-HODE, pmol/ml	20.2 ± 5.8	21.5 ± 7.7	22.8 ± 6.3	25.1 ± 8.4
hsCRP, μg/ml	0.77 (0.36–1.38)	0.75 (0.39–1.54)	0.82 (0.43–1.74)	0.91 (0.48–1.93)
IL-6, pg/ml	3.05 ± 1.53	3.41 ± 1.75	4.08 ± 1.66	4.43 ± 1.97
MCP-1, pg/ml	227.3 ± 74.5	269.1 ± 92.8	294.8 ± 82.3	334.7 ± 112.6
TNF-α, ng/ml	2.34 ± 0.68	2.55 ± 0.76	2.87 ± 0.82	3.19 ± 1.03

^1^Mean ± SD (all such values).

^2^Includes coronary heart disease, stroke, hypertension, and diabetes in a parent or a first-degree sibling.

^3^Median (interquartile range).

There were significant correlations between plasma RBP4 and plasma fasting glucose (*P* = 0.018) and HOMA-IR (*P* = 0.048), but no significant associations with insulin were found (*P* = 0.054, Table 2). The participants in the highest quartile of plasma RBP4 levels had higher plasma fasting glucose concentrations than those in the lower quartiles. The plasma RBP4 levels showed a strong and positive correlation with triglycerides (*r* = 0.272, *P* < 0.001). A higher plasma RBP4 was significantly correlated with both higher LDL cholesterol (*P* < 0.001) and lower HDL cholesterol (*P* = 0.004). No significant associations were observed for blood pressure. RBP4 was also significantly associated with elevated A-FABP levels (*r* = 0.104, *P* = 0.001), but no correlation was observed between RBP4 and adiponectin (*r* = 0.030, *P* = 0.151). In addition, RBP4 was strongly correlated with not only oxidative markers, such as 8-iso PGF2α (*r* = 0.236, *P* < 0.001) and 13-HODE (*r* = 0.204, *P* < 0.001), but also with elevated proinflammatory molecules, including hsCRP (*r* = 0.118, *P* = 0.011), IL6 (*r* = 0.236, *P* < 0.001), MCP1 (*r* = 0.145, *P* = 0.005) and TNFα (*r* = 0.187, *P* < 0.001).

**Table 2:** **Multivariable-adjusted Spearman correlation coefficients of RBP4 and metabolic risk factors**^**1**^

	**Spearman correlation coefficient**	***P***
BMI	0.108	0.004
Systolic blood pressure	0.034	0.235
Diastolic blood pressure	0.042	0.189
Fasting glucose	0.095	0.018
Insulin	0.046	0.054
HOMA-IR	0.052	0.048
Adiponectin	0.030	0.151
A-FABP	0.104	<0.001
Total cholesterol	0.139	<0.001
Triglycerides	0.272	<0.001
HDL cholesterol	−0.072	0.004
LDL cholesterol	0.144	<0.001
8-iso PGF2α	0.236	<0.001
13-HODE	0.204	<0.001
hsCRP	0.118	0.011
IL6	0.236	<0.001
MCP1	0.145	0.005
TNFα	0.187	<0.001

^1^Spearman correlation coefficients were adjusted by age, sex and BMI. A-FABP, adipocyte fatty acid-binding protein; 13-HODE, 8-iso PGF2α, 8-iso-prostaglandin F2α; 13-(S)-hydroxyoctadecadienoic acid (13-HODE); hsCRP, high sensitivity C-reactive protein; RBP-4, retinol-binding protein 4.

**Figure 1:**
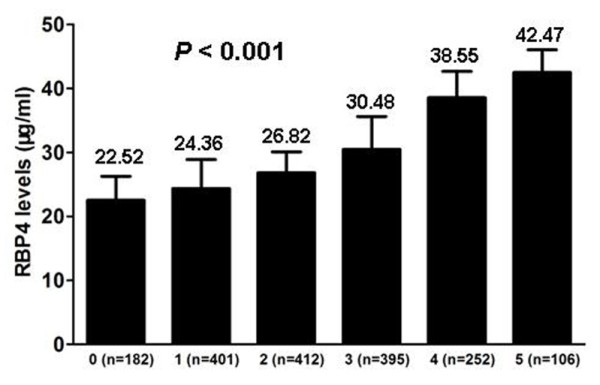
**Plasma RBP4 concentrations according to the number of metabolic syndrome components.***P* values were calculated from the multivariable-adjusted general linear regression model. The covariates adjusted included age, sex, drinking, smoking, family history of diabetes and cardiovascular diseases.

The RBP4 levels increased gradually with the number of MetS components (Figure 1). The RBP4 levels increased from 22.52 μg/ml in the participants with none of the MetS components up to 42.47 μg/ml in those with all 5 components. The OR (95% CI) for MetS was 3.46 (2.87, 4.42) in the highest quartile of RBP4 compared with the lowest quartile, after controlling for age, sex, BMI, family history of chronic diseases and self-reported chronic diseases (Table 3). RBP4 was also positively associated with some of the MetS components, including hypertriglyceridemia, reduced HDL cholesterol, elevated LDL cholesterol and hyperglycemia. These associations were not materially attenuated by further adjustment of adipokines and oxidative and inflammatory markers.

**Table 3:** **OR (and 95% CI) of MetS and its components according to RBP4 quartiles**^**1**^

	**RBP4 quartiles**				
	**1**	**2**	**3**	**4**	***P***^**2**^
Metabolic syndrome (no. cases)	131	171	193	251	
Model 1^3^	1.00	1.65 (1.29, 1.99)	2.28 (1.76, 2.89)	3.84 (3.12, 4.58)	<0.001
Model 2^4^	1.00	1.61 (1.26, 1.97)	2.15 (1.65, 2.73)	3.46 (2.87, 4.42)	<0.001
Model 3^5^	1.00	1.53 (1.21, 1.92)	2.04 (1.58, 2.47)	2.68 (1.95, 3.67)	<0.001
Central obesity	183	204	214	331	
Model 1	1.00	1.23 (1.02, 1.51)	1.42 (1.08, 1.75)	1.87 (1.48, 2.36)	<0.001
Model 2	1.00	1.01 (0.74, 1.32)	0.97 (0.67, 1.28)	0.92 (0.66, 1.35)	0.69
Model 3	1.00	0.94 (0.66, 1.29)	0.87 (0.65, 1.25)	0.81 (0.61, 1.14)	0.23
Hypertriglyceridemia	46	77	112	197	
Model 1	1.00	2.13 (1.64, 2.87)	3.45 (2.61, 4.49)	6.75 (5.28, 8.96)	<0.001
Model 2	1.00	2.06 (1.52, 2.83)	3.26 (2.47, 4.33)	5.92 (4.47, 8.02)	<0.001
Model 3	1.00	1.97 (1.46, 2.68)	3.01 (2.16, 4.08)	5.03 (3.59, 7.45)	<0.001
Reduced HDL cholesterol	154	171	177	186	
Model 1	1.00	1.24 (1.05, 1.51)	1.38 (1.15, 1.67)	1.76 (1.26, 2.17)	<0.001
Model 2	1.00	1.15 (0.97, 1.39)	1.27 (1.04, 1.58)	1.58 (1.17, 1.84)	<0.001
Model 3	1.00	1.07 (0.90, 1.26)	1.18 (0.96, 1.51)	1.42 (1.11, 1.68)	<0.001
Elevated blood pressure	161	116	117	132	
Model 1	1.00	0.93 (0.81, 1.17)	1.02 (0.84, 1.33)	1.10 (0.95, 1.44)	0.32
Model 2	1.00	0.87 (0.76, 1.15)	0.97 (0.81, 1.24)	1.02 (0.84, 1.29)	0.11
Model 3	1.00	0.83 (0.68, 1.10)	0.76 (0.62, 1.02)	0.73 (0.61, 1.16)	0.08
Hyperglycemia	135	167	188	245	
Model 1	1.00	1.57 (1.26, 1.95)	1.72 (1.35, 2.14)	3.13 (2.44, 3.93)	<0.001
Model 2	1.00	1.50 (1.17, 1.84)	1.67 (1.31, 1.96)	2.74 (2.15, 3.36)	<0.001
Model 3	1.00	1.42 (1.06, 1.75)	1.52 (1.23, 1.75)	2.32 (1.85, 2.91)	<0.001

^1^Metabolic syndrome was defined according to the updated National Cholesterol Education Program Adult Treatment Panel III criteria for Asian Americans.

^2^Calculated by using multivariable logistic regression.

^3^Adjusted for age and sex.

^4^Adjusted as for model 1 plus BMI (except when modeling associations for central obesity), smoking, drinking, family history of cardiovascular diseases and diabetes.

^5^Adjusted as for model 2 plus adiponectin, A-FABP, 8-iso PGF2α, 13-HODE, hsCRP, IL6, MCP1 and TNFα.

## Discussion

In the present study, we observed strong positive associations between plasma RBP4 concentrations and the increased risk of MetS and its components among middle-aged and elderly Chinese people. Furthermore, a chronic inflammatory response was significantly associated with higher plasma RBP4 levels, and the participants with those conditions were more likely to have higher RBP4 levels. Additionally, higher RBP4 levels were associated with oxidative stress, as reflected by a higher concentration of the oxidative markers.

RBP4, which is mainly secreted from adipocytes and hepatocytes, has been suggested to be a central regulator of insulin sensitivity. In animal models, the overexpression of human RBP4 or the injection of recombinant RBP4 induces insulin resistance in mice, whereas RBP4 knockout mice showed enhanced insulin sensitivity [23]. Our results suggest that high plasma RBP4 levels were associated with higher fasting glucose levels, Hb Alc and HOMA index, which was consistent with the animal studies. Furthermore, we also observed a positive association between plasma RBP4 and total cholesterol, LDL cholesterol and triglycerides, but we observed an inverse correlation with HDL cholesterol concentrations in this population. We postulate that the effect of RBP4 on blood lipid concentrations, especially triglycerides, may be mediated through its effect on the metabolism of hepatic fatty acids, which regulates the expression of genes involved in lipid metabolism [24].

In humans, several studies have also shown positive associations between RBP4 and insulin resistance as well as features of MetS [25,26]. However, several subsequent studies have failed to confirm these associations [27,28]. In the current study, we found positive associations of RBP4 with traditional metabolic traits and an increased risk of MetS presence. High levels of A-FABP and CRP and low levels of adiponectin are the well-documented risk factors for MetS and its components [29-31]. However, the relationship between RBP4 and A-FABP, CRP or adiponectin has not been well addressed because of the controversial reports. Balagopal *et al*. [32] reported that RBP4 was positively associated with CRP and negatively associated with adiponectin in a small group of obese children; however, no correlation between RBP4 and CRP or adiponectin was observed in another study of 101 hospitalized T2DM patients [33]. With a larger sample size, Qi Q [25] demonstrated a weak inverse correlation between RBP4 and adiponectin levels, but no correlation was detected between RBP4 and CRP levels. In this study, we indeed found a moderate inverse correlation between RBP4 and CRP levels, but no correlation was observed between RBP4 and adiponectin. Interestingly, we observed a positive association between RBP4 and A-FABP. RBP4 and A-FABP are two members of the lipocalin family, which are produced from mature adipocytes. We hypothesize that adipose tissues secrete these two adipocytokines via the same mechanism. More importantly, increased RBP4 per se was an independent risk factor for MetS, even within the lowest A-FABP or CRP quartile or the highest adiponectin quartile. Therefore, it is possible that RBP4 may promote MetS risk through a distinct pathway that does not fully overlap with F-FABP, CRP or adiponectin.

The obese condition and MetS are accompanied by low-grade chronic inflammation, which is characterized by an increased expression of inflammatory cytokines and infiltration of immune cells in adipocytes [34]. The inflammatory response promotes the activation of transcriptional factors and pro-inflammatory cytokines, which can lead to an unresolved inflammatory response associated with an inhibition of insulin signaling and a high risk for cardiovascular events [35]. In the present study, we observed a strong correlation between RBP4 levels and elevated inflammatory markers, including IL-6, MCP-1 and TNF-α; this correlation has been well documented to play an important role in the initiation and development of inflammatory effects in adipose tissue. Thus, our results identified proinflammatory markers may be a critical link between RBP4 and the pathogenesis of MetS.

Accumulating evidence also supports the important role that oxidative stress plays in MetS-related manifestations [36]. In this study, higher plasma RBP4 levels were significantly associated with higher serum 8-iso PGF2-α and 13-HODE, which suggests that a higher RBP4 status may contribute to oxidative damage. Both 8-iso PGF2-α and 13-HODE are major products of the peroxidation of unsaturated fatty acids and were shown to be reliable markers for oxidative stress [37,38]. Thus, the association of RBP4 with systematic oxidative stress markers in humans may explain the possible mechanism of a high RBP4 status on metabolic disorders in humans.

Several limitations of this study need to be addressed. First, the cross-sectional nature of the study design cannot be translated into a clear cause–effect inference. Prospective studies and randomized clinical trials are needed. Second, as with any observational study, the role of unmeasured or residual confounding cannot be ruled out; however, the multivariate models did adjust for a wide range of risk factors that have been implicated in the development of MetS or its individual components. Finally, we did not distinguish among full-length and truncated forms of RBP4 that might have various biological activities with potential varying effects on metabolic risk factors and MetS. Future studies with multiple measurements may substantiate our findings.

In conclusion, there was a strong positive association between plasma RBP4 with the risk of developing MetS in a middle-aged and elderly Chinese population: subjects with MetS or its components had a higher RBP4 status than those without these conditions. Moreover, the participants with high plasma RBP4 levels were also associated with the inflammatory markers and oxidative stress. Our results suggest a potential link between RBP4 and the incidence of MetS.

## Abbreviations

BMI: Body-mass index; CI: Confidence intervals; CRP: High-sensitivity C-reactive protein; CVD: Cardiovascular disease; HDL-C: High-density lipoprotein cholesterol; LDL-C: Low-density lipoprotein cholesterol; 8-iso PGF2α: 8-iso-prostaglandin F2α; MetS: Metabolic syndrome; MCP1: Monocyte chemotactic protein-1; OR: Odds ratio; RBP4: Retinol-binding protein 4; TG: Triglyceride; T2DM: Type 2 diabetes mellitus; WHR: Waist-to-hip ratio.

## Competing interests

The authors declare that they have no competing interests.

## Authors’ contributions

YL and DW conducted the research, performed the statistical analyses and wrote the manuscript; YL, DW and MW participated in the data collection and checked the data; and MX participated in the design of this study and wrote the manuscript. All authors have read and approved the final manuscript.

## Relationships with industry

None of the authors have relationships with industry.

## References

[B1] Ford ES Giles WH Dietz WH Prevalence of the metabolic syndrome among US adults: findings from the third National Health and Nutrition Examination Survey JAMA 2002 287 356 359 10.1001/jama.287.3.356 11790215

[B2] Expert Panel on Detection, Evaluation, and Treatment of High Blood Cholesterol in Adults Executive summary of the third report of the National Cholesterol Education Program (NCEP) expert panel on detection, evaluation, and treatment of high blood cholesterol in adults (adult treatment panel III) JAMA 2001 285 2486 2497 10.1001/jama.285.19.2486 11368702

[B3] Eckel RH Grundy SM Zimmet PZ The metabolic syndrome Lancet 2005 365 1415 1428 10.1016/S0140-6736(05)66378-7 15836891

[B4] Hee Park K Zaichenko L Brinkoetter M Thakkar B Sahin-Efe A Joung KE Tsoukas MA Geladari EV Huh JY Dincer F Davis CR Crowell JA Mantzoros CS Circulating irisin in relation to insulin resistance and the metabolic syndrome J Clin Endocrinol Metab 2013 98 4899 4907 10.1210/jc.2013-2373 24057291 PMC3849667

[B5] Maury E Noël L Detry R Brichard SM In vitro hyperresponsiveness to tumor necrosis factor-alpha contributes to adipokine dysregulation in omental adipocytes of obese subjects J Clin Endocrinol Metab 2009 94 1393 1400 10.1210/jc.2008-2196 19174496

[B6] Han TS Sattar N Williams K Gonzalez-Villalpando C Lean MEJ Haffner SM Prospective study of C-reactive protein in relation to the development of diabetes and metabolic syndrome in the Mexico City Diabetes Study Diabetes Care 2002 25 2016 2021 10.2337/diacare.25.11.2016 12401749

[B7] Furukawa S Fujita T Shimabukuro M Iwaki M Yamada Y Nakajima Y Nakayama O Makishima M Matsuda M Shimomura I Increased oxidative stress in obesity and its impact on metabolic syndrome J Clin Invest 2004 114 1752 1761 10.1172/JCI21625 15599400 PMC535065

[B8] Blaner WS Retinol-binding protein: the serum transport protein for vitamin A Endocr Rev 1989 10 308 316 10.1210/edrv-10-3-308 2550213

[B9] Graham TE Yang Q Blüher M Hammarstedt A Ciaraldi TP Henry RR Wason CJ Oberbach A Jansson PA Smith U Kahn BB Retinol-binding protein 4 and insulin resistance in lean, obese, and diabetic subjects N Engl J Med 2006 354 2552 2563 10.1056/NEJMoa054862 16775236

[B10] Norseen J Hosooka T Hammarstedt A Yore MM Kant S Aryal P Kiernan UA Phillips DA Maruyama H Kraus BJ Usheva A Davis RJ Smith U Kahn BB Retinol-binding protein 4 inhibits insulin signaling in adipocytes by inducing proinflammatory cytokines in macrophages through a c-Jun N-terminal kinase- and toll-like receptor 4-dependent and retinol-independent mechanism Mol Cell Biol 2012 32 2010 2019 10.1128/MCB.06193-11 22431523 PMC3347417

[B11] Klöting N Graham TE Berndt J Kralisch S Kovacs P Wason CJ Fasshauer M Schön MR Stumvoll M Blüher M Kahn BB Serum retinol-binding protein is more highly expressed in visceral than in subcutaneous adipose tissue and is a marker of intra-abdominal fat mass Cell Metab 2007 6 79 87 10.1016/j.cmet.2007.06.002 17618858

[B12] Tschoner A Sturm W Engl J Kaser S Laimer M Laimer E Weiss H Patsch JR Ebenbichler CF Retinol-binding protein 4, visceral fat, and the metabolic syndrome: effects of weight loss Obesity (Silver Spring) 2008 16 2439 2444 10.1038/oby.2008.391 18719670

[B13] Ng TW Watts GF Barrett PH Rye KA Chan DC Effect of weight loss on LDL and HDL kinetics in the metabolic syndrome: associations with changes in plasma retinol-binding protein-4 and adiponectin levels Diabetes Care 2007 30 2945 2950 10.2337/dc07-0768 17686833

[B14] Ingelsson E Sundström J Melhus H Michaëlsson K Berne C Vasan RS Risérus U Blomhoff R Lind L Arnlöv J Circulating retinol-binding protein 4, cardiovascular risk factors and prevalent cardiovascular disease in elderly Atherosclerosis 2009 206 239 244 10.1016/j.atherosclerosis.2009.02.029 19339013

[B15] Sun Q Kiernan UA Shi L Phillips DA Kahn BB Hu FB Manson JE Albert CM Rexrode KM Plasma retinol-binding protein 4 (RBP4) levels and risk of coronary heart disease: a prospective analysis among women in the nurses’ health study Circulation 2013 127 1938 1947 10.1161/CIRCULATIONAHA.113.002073 23584360 PMC3741657

[B16] Cho YM Youn BS Lee H Lee N Min SS Kwak SH Lee HK Park KS Plasma retinol-binding protein-4 concentrations are elevated in human subjects with impaired glucose tolerance and type 2 diabetes Diabetes Care 2006 29 2457 2461 10.2337/dc06-0360 17065684

[B17] Chavez AO Coletta DK Kamath S Cromack DT Monroy A Folli F DeFronzo RA Tripathy D Retinol-binding protein 4 is associated with impaired glucose tolerance but not with whole body or hepatic insulin resistance in Mexican Americans Am J Physiol Endocrinol Metab 2009 296 E758 E764 10.1152/ajpendo.90737.2008 19190263

[B18] Kotnik P Fischer-Posovszky P Wabitsch M RBP4—a controversial adipokine Eur J Endocrinol 2011 165 703 711 10.1530/EJE-11-0431 21835764

[B19] Promintzer M Krebs M Todoric J Luger A Bischof MG Nowotny P Wagner O Esterbauer H Anderwald C Insulin resistance is unrelated to circulating retinol binding protein and protein C inhibitor J Clin Endocrinol Metab 2007 92 4306 4343 sss12 10.1210/jc.2006-2522 17726077

[B20] Graham TE Wason CJ Bluher M Kahn BB Shortcomings in methodology complicate measurements of serum retinol binding protein (RBP4) in insulin-resistant human subjects Diabetologia 2007 50 814 823 10.1007/s00125-006-0557-0 17294166

[B21] Friedewald WT Levy RI Fredrickson DS Estimation of the concentration of low-density lipoprotein cholesterol in plasma, without use of the preparative ultracentrifuge Clin Chem 1972 18 499 502 4337382

[B22] Grundy SM Cleeman JI Daniels SR Donato KA Eckel RH Franklin BA Gordon DJ Krauss RM Savage PJ Smith SC Jr Spertus JA Costa F Diagnosis and management of the metabolic syndrome: an American Heart Association/National Heart, Lung, and Blood Institute Scientific Statement Circulation 2005 112 2735 2752 10.1161/CIRCULATIONAHA.105.169404 16157765

[B23] Yang Q Graham TE Mody N Preitner F Peroni OD Zabolotny JM Kotani K Quadro L Kahn BB Serum retinol binding protein 4 contributes to insulin resistance in obesity and type 2 diabetes Nature 2005 436 356 362 10.1038/nature03711 16034410

[B24] Xia M Liu Y Guo H Wang D Wang Y Ling W Retinol binding protein 4 stimulates hepatic sterol regulatory element-binding protein 1 and increases lipogenesis through the peroxisome proliferator-activated receptor-γ coactivator 1β-dependent pathway Hepatology 2013 58 564 575 23300015 10.1002/hep.26227

[B25] Qi Q Yu Z Ye X Zhao F Huang P Hu FB Franco OH Wang J Li H Liu Y Lin X Elevated retinol-binding protein 4 levels are associated with metabolic syndrome in Chinese people J Clin Endocrinol Metab 2007 92 4827 4834 10.1210/jc.2007-1219 17878249

[B26] Hammarstedt A Graham TE Kahn BB Adipose tissue dysregulation and reduced insulin sensitivity in non-obese individuals with enlarged abdominal adipose cells Diabetol Metab Syndr 2012 4 42 10.1186/1758-5996-4-42 22992414 PMC3523053

[B27] Takashima N Tomoike H Iwai N Retinol-binding protessin 4 and insulin resistance N Engl J Med 2006 355 1392 author reply 1394–139s5 17005964 10.1056/NEJMc061863

[B28] Janke J Engeli S Boschmann M Adams F Böhnke J Luft FC Sharma AM Jordan J Retinol-binding protein 4 in human obesity Diabetes 2006 55 2805 2810 10.2337/db06-0616 17003346

[B29] Matsuzawa Y Funahashi T Kihara S Shimomura I Adiponectin and metabolic syndrome Arterioscler Thromb Vasc Biol 2004 24 29 33 10.1161/01.ATV.0000099786.99623.EF 14551151

[B30] Xu A Tso AW Cheung BM Wang Y Wat NM Fong CH Yeung DC Janus ED Sham PC Lam KS Circulating adipocyte-fatty acid binding protein levels predict the development of the metabolic syndrome: a 5-year prospective study Circulation 2007 115 1537 1543 10.1161/CIRCULATIONAHA.106.647503 17389279

[B31] Ye X Yu Z Li H Franco OH Liu Y Lin X Distributions of C-reactive protein and its association with metabolic syndrome in middle-aged and older Chinese people J Am Coll Cardiol 2007 49 1798 1805 10.1016/j.jacc.2007.01.065 17466231

[B32] Balagopal P Graham TE Kahn BB Altomare A Funanage V George D Reduction of elevated serum retinol binding protein in obese children by lifestyle intervention: association with subclinical inflammation J Clin Endocrinol Metab 2007 92 1971 1974 10.1210/jc.2006-2712 17341558

[B33] Takebayashi K Suetsugu M Wakabayashi S Aso Y Inukai T Retinol binding protein-4 levels and clinical features of type 2 diabetes patients J Clin Endocrinol Metab 2007 92 2712 2719 10.1210/jc.2006-1249 17440021

[B34] Sell H Habich C Eckel J Adaptive immunity in obesity and insulin resistance Nat Rev Endocrinol 2012 8 709 716 10.1038/nrendo.2012.114 22847239

[B35] Romeo GR Lee J Shoelson SE Metabolic syndrome, insulin resistance, and roles of inflammation–mechanisms and therapeutic targets Arterioscler Thromb Vasc Biol 2012 32 1771 1776 10.1161/ATVBAHA.111.241869 22815343 PMC4784686

[B36] Elnakish MT Hassanain HH Janssen PM Angelos MG Khan M Emerging role of oxidative stress in metabolic syndrome and cardiovascular diseases: important role of Rac/NADPH oxidase J Pathol 2013 231 290 300 24037780 10.1002/path.4255

[B37] Pratico D F(2)-isoprostanes: sensitive and specific non-invasive indices of lipid peroxidation in vivo Atherosclerosis 1999 147 1 10 10.1016/S0021-9150(99)00257-9 10525118

[B38] Vangaveti V Baune BT Kennedy RL Hydroxyoctadecadienoic acids: novel regulators of macrophage differentiation and atherogenesis Ther Adv Endocrinol Metab 2010 1 51 60 10.1177/2042018810375656 23148150 PMC3475286

